# Development of a simple, rapid, and sensitive diagnostic assay for enterotoxigenic *E*. *coli* and *Shigella spp* applicable to endemic countries

**DOI:** 10.1371/journal.pntd.0010180

**Published:** 2022-01-28

**Authors:** Subhra Chakraborty, Sean Connor, Mirza Velagic

**Affiliations:** Department of International Health, Johns Hopkins Bloomberg School of Public Health, Baltimore, Maryland, United States of America; University of Washington, UNITED STATES

## Abstract

Enterotoxigenic *E*. *coli* (ETEC) and *Shigella spp* (Shigella) are complex pathogens. The diagnostic assays currently used to detect these pathogens are elaborate or complicated, which make them difficult to apply in resource poor settings where these diseases are endemic. The culture methods used to detect Shigella are not sensitive, and the methods used to detect ETEC are only available in a few research labs. To address this gap, we developed a rapid and simple diagnostic assay–"Rapid LAMP based Diagnostic Test (RLDT)." The six minutes sample preparation method directly from the fecal samples with lyophilized reaction strips and using established Loop-mediated Isothermal Amplification (LAMP) platform, ETEC [heat labile toxin (LT) and heat stable toxins (STh, and STp) genes] and Shigella (*ipaH* gene) detection was made simple, rapid (<50 minutes), and inexpensive. This assay is cold chain and electricity free. Moreover, RLDT requires minimal equipment. To avoid any end user’s bias, a battery-operated, handheld reader was used to read the RLDT results. The results can be read as positive/negative or as real time amplification depending on the end user’s need. The performance specifications of the RLDT assay, including analytical sensitivity and specificity, were evaluated using fecal samples spiked with ETEC and Shigella strains. The limit of detection was ~10^5^ CFU/gm of stool for LT, STh, and STp and ~10^4^ CFU/gm of stool for the *ipaH* gene, which corresponds to about 23 CFU and 1 CFU respectively for ETEC and Shigella per 25uL reaction within 40 minutes. The RLDT assay from stool collection to result is simple, and rapid and at the same time sufficiently sensitive. RLDT has the potential to be applied in resource poor endemic settings for the rapid diagnosis of ETEC and Shigella.

## Author summary

Enterotoxigenic *E*. *coli* and *Shigella* are the leading causes of moderate to severe diarrhea in the low-and middle-income countries (LMICs). A critical constraint to determine the ETEC and Shigella disease burden at the country or sub-national level, is the complex diagnostic methods currently required for detecting these pathogens. These methods are neither sufficiently sensitive nor standardized and are not feasible in the resource poor settings where these infections occur most commonly. We developed a simple and rapid diagnostic assay called "Rapid Loop-mediated isothermal amplification based Diagnostic Test (RLDT)" for the detection of these pathogens in low-resource settings. Using RLDT, ETEC and Shigella were detected directly from the stool, in less than 1 hour with minimal hands-on time. The assay does not require maintaining a cold chain and is electricity-free. Being rapid, simple, and sensitive, RLDT can be scaled up and is appropriate to apply in the LMICs where ETEC and Shigella diarrhea are endemic.

## Introduction

Enterotoxigenic *E*. *coli* (ETEC) and *Shigella spp* (Shigella) are the primary causes of moderate-to-severe diarrhea (MSD) in children <5 years of age living in impoverished areas of the world [1,2]. These pathogens are also the most frequent bacterial causes of diarrhea among travelers to Africa, Asia, and Latin America, including military personnel deployed to these areas [3–4].

ETEC and Shigella are complex pathogens, and the diagnostic assays used to detect these pathogens are either elaborate or complex. ETEC are characterized on a molecular basis by the presence of genes that encode the heat-stable (ST) and heat-labile (LT) enterotoxins [1,5]. In the absence of selective media, the most frequently used diagnostic assay for ETEC is culturing the stool samples on culture media like MacConkey agar and isolating 3 to 5 *E*. *coli* like colonies, followed by PCR targeting the toxin genes [1,2,5,6,7]. The other diagnostic methods used are GM1 ganglioside ELISA and DNA probe hybridization assays, targeting the toxins using *E*. *coli* colonies [1,8]. A conventional bacterial culture is a gold standard for the detection of Shigella [2,9,10]. For detection of Shigella, stool specimens are inoculated onto selective culture media like Xylose Lysine Deoxycholate (XLD), Hektoen Enteric agar (HEA), and Salmonella Shigella Agar (SSA) [10] and Shigella-like colonies are then selected for further biochemical analysis and confirmed serologically by slide agglutination using commercially available antisera [2,10]. Recent studies have shown that the current diagnostic methods for ETEC and Shigella are not sufficiently sensitive to reflect the true burden of these pathogens. The sensitivity of these assays depends on the number of *E*. *coli* colonies or suspected Shigella colonies screened [11–13].

An alternative diagnostics approach is quantitative PCR (qPCR) which is highly sensitive and can significantly increase the diagnostic yield [7,11–13]. qPCR generally targets the toxin genes for ETEC and invasion plasmid gene (*ipaH*) for Shigella for detection of these pathogens [7,11–13]. However, qPCR, along with other current diagnostic methods, are technology and equipment dependent, require extensive sample preparation protocols, and a cold chain. In addition, qPCR require more sophisticated laboratory support facilities; more extensively trained personnel and are time-consuming and expensive. These methods are a better fit for centralized labs and are challenging to apply in resource poor settings where ETEC and Shigella infections are endemic. Therefore, a diagnostic method that is simple, rapid, inexpensive as well as sufficiently sensitive is required for the detection of ETEC and Shigella [14,15].

To address this technology gap in the ETEC and Shigella diagnostics, we developed a simple, rapid, cold chain and electricity free diagnostic test “Rapid Loop-mediated isothermal amplification-based Diagnostic Test (RLDT)”. The analytical performance specification tests of RLDT kit are also described in this manuscript.

We believe this assay has the potential to be more applicable for ETEC and Shigella diagnosis in low-and middle-income countries (LMICs) and we feel the encouraging results presented here justify further test evaluation and validation.

## Materials and methods

### Optimization of ETEC and Shigella RLDT assay

The RLDT assay is based on the LAMP technology, modified to be applicable to the resource poor settings. The LAMP is a nucleic acid amplification method under isothermal conditions, first developed by Notomi *et al* [16]. The LAMP technique is based on auto cycling and high DNA strand displacement activity through a repetition of two types of elongation reactions occurring at the loop regions: self-elongation of templates from the stem loop structure formed at the 3-terminal and the binding and elongation of new primers to the loop region [16].

The targets for RLDT were selected as LT, STh, and STp genes to detect ETEC and invasion plasmid gene (*ipaH*) for Shigella. The primers were designed using the online software Primer Explorer V5 (https://primerexplorer.jp/e/). A set of 3 primer pairs, including two outer primers (forward primer F3 and backward primer B3), two inner primers (forward inner primer FIP and backward inner primer BIP), and two loop primers (forward loop primer LF and backward loop primer LB), were selected. The primers were assessed for specificity using the Basic Local Alignment Search Tool (BLAST) of the National Center for Biotechnology Information (NCBI) against sequences in GenBank. The primer sequences, concentrations, and GenBank IDs are shown in Table 1.

**Table 1:** List of ETEC and Shigella RLDT primers used in this study.

**ETEC LT**	GenBank: FN649414.1		
**Primer Name**	**Sequence (5’-3’)**	**Primer Concentration**	**Reference**
F3	ATCGTGTTAATTTTGGTGTGATTG	0.8 μM	This Study
B3	CTGGGTCTCCTCATTACAAGT	0.8 μM	
FIP	AACCATCCTCTGCCGGAGCTATATTGAACGATTACATCGTAACAGGGAAT	1.6 μM	
BIP	TTCCCACCGGATCACCAAGCTGTTCTTGATGAATCTCCACAACCTT	1.6 μM	
LF	GATTTCTGTAATACCGGTCTCTAT	0.8 μM	
LB	AGAAGAACCCTGGATTCATCATGC	0.8 μM	
**ETEC STp**	GenBank: FN649414.1		
**Primer Name**	**Sequence (5’-3’)**	**Primer Concentration**	**Reference**
F3	GCAAAATCCGTTTAACTAATCTCAA	0.8 μM	This Study
B3	ACAGCAGTAAAATGTGTTGTTCAT	0.8 μM	
FIP	AAGAGGGGAAAGATAATACAGAAATTTTTTAAACAACATGACGGGAGGT	1.6 μM	
BIP	TAGTCAGTCAACTGAATCACTTGATTTTTCTGTTGTTTTTTACAACATCACACT	1.6 μM	
LF	GCCAACATTAGCTTTTTCATG	0.8 μM	
LB	CAAAAGAGAAAATTACATTAGAGAC	0.8 μM	
**ETEC STh**	GenBank: FN649414.1		
**Primer Name**	**Sequence (5’-3’)**	**Primer Concentration**	**Reference**
F3	CTCAGGATGCTAAACCAGT	0.2 μM	Yano et al. 2007 [17]
B3	CAGAACAAATATAAAGGGAACTGTT	0.2 μM	
FIP	TCATGCTTTCAGGACCACTTTTATTGAGTCTTCAAAAGAAAAAATCACACT	1.6 μM	
BIP	AGTAGCAATTACTGCTGTGAATTGTCCCTTTATATTATTAATAGCACCCG	1.6 μM	
LB	GTTGTAATCCTGCTTGT	0.8 μM	
**Shigella *ipaH***	GenBank: M76444.1		
**Primer Name**	**Sequence (5’-3’)**	**Primer Concentration**	**Reference**
F3	AATTCTGGAGGACATTGCC	0.2 μM	This Study
B3	CGTACGCTTCAGTACAGC	0.2 μM	
FIP	CTTCACGGCAGTGGAGAGCTGAGATAGAAGTCTACCTGGC	1.6 μM	
BIP	TATGGCGTGTCGGGAGTGATTCATTCTCTTCACGGCTTC	1.6 μM	
LF	CTCTGCGAGCATGGTCTG	0.8 μM	
LB	CACTGCCGAAGCTATGGT	0.8 μM	

A loop-mediated isothermal amplification (LAMP) based assay was developed to detect ETEC and *Shigella* using OmniAmp 2X Isothermal Master Mix (Lucigen, WI). The final concentrations of the reaction mixtures were 1X OmniAmp Master Mix, 2 mM FionaGreen dye (Marker Gene, OR), 1X LAMP primer mix, and 5 μL of DNA, brought to volume (25 μL) with DNase-RNase–free water. Initial optimization experiments were performed on a Step One Plus Real-Time PCR System (Applied Biosystems, CA). Amplification was monitored by the detection of Fiona Green fluorescence and quantified by the instrument software. To calculate the time to result (TTR), the threshold was set as 3000 or 4000 relative fluorescence units (RFU). To determine optimum temperature, reactions were performed using ETEC and Shigella strains at temperatures between 68°C to 74°C in 1°C increment for 30 to 60 minutes in 10 minutes increments. Later experiments were performed using a real-time isothermal fluorometer (reader) (AmpliFire, Agdia Inc., IN). The ETEC primers were tested for any cross-reactivity against the *ctxA* gene of *Vibrio cholerae*. The *ipaH* primers were tested against *S*. *flexneri*, *S*. *sonnei*, *S*. *dysenteriae*, and *S*. *boydii* to confirm that these primers can detect *Shigella spp* and serogroups.

**Fig 1:**
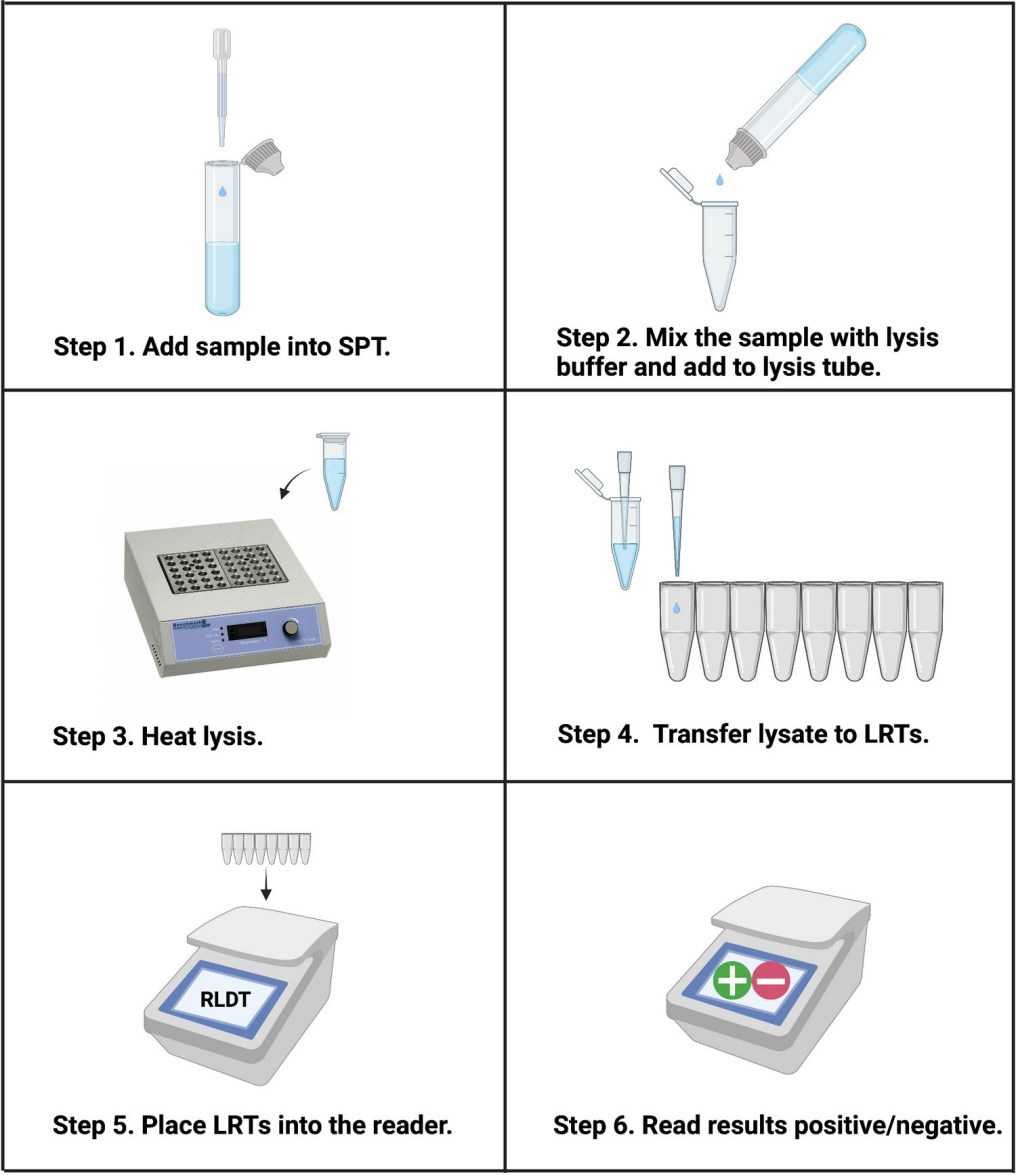
Workflow of ETEC and Shigella RLDT using RLDT kit. Step 1, using the fixed volume pipette provided in the kit, add a stool sample to the Sample Processing Tube (SPT) with lysis buffer. Step 2, Cap the SPT tube and mix by up and down. Squeeze the SPT tube to add the lysis buffer + stool sample to the lysis tube (1.5ml microfuge tube). Step 3, lysis tube is heated at 90°C for 5 minutes in a heat block or water bath. Step 4, add 25 μL of lysate to each tube of the lyophilized reaction tubes (LRT) strip using disposable fixed volume pipette. Step 5, place the LRTs in the reader. Scan the barcode for ETEC and Shigella RLDT program. Step 6, read results displayed on the screen after 40 minutes. All the reagents and sample processing accessories required for RLDT are included in the kit. Illustration was created with BioRender.com.

To make RLDT assay appropriate to use in the low resource settings, a simple and rapid sample preparation method directly from the stool samples was developed. Heat lysis was performed following dilution of stool sample into an extraction lysis buffer using a sample preparation tube (SPT) (Fig 1). M13mp18 plasmid (New England BioLabs, MA) was included as the assay inhibitor control in the extraction buffer. The lysate was used as a template in RLDT reactions.

To allow ambient storage, a complete 1X OmniAmp-LAMP formulation and 10% trehalose (Sigma-Aldrich, MO) was dispensed into 0.2ml microfuge tubes in lyophilized reaction tube (LRT) strips. The mixture was lyophilized at the JHU core laboratory using a Labconco FreeZone bench-top lyophilizer (Labconco Corporation, MO). The LRT strips were capped and packed in light-resistant bags with a desiccant pouch.

LRT strips were rehydrated with sample lysate to a total volume of 25μl per tube and placed in the reader for 40 minutes to detect the target genes of ETEC and Shigella (Fig 1).

### ETEC and Shigella RLDT kit

The RLDT assay is developed as a kit. The kit includes dry RLDT LRT strips and accessories for sample preparation. Each LRT strip has eight microfuge tubes. One LRT strip per sample was designed as duplicate microfuge tubes filled with the RLDT complete master mix targeting LT, STh, and *ipaH* genes, with one target in each tube and STp as a single tube. In addition, a tube for reaction inhibitor control was added in duplicates per sample. This arrangement of the targets is flexible and could be changed depending on the requirement and purpose of the applications.

The fluorometer reader that was used in this study can incubate and read one LRT strip, eight targets simultaneously. This device is optimized for isothermal chemistry and allows real-time monitoring of amplification. It offers a touch screen interface, data storage, and portability (handheld) with a rechargeable battery. The algorithm can be set depending on the end user’s need for binary +/- results or in-depth analysis of the real time amplification. The RLDT assay programs are coded in bar codes and scanned to include in the reader.

### Performance testing of ETEC and Shigella RLDT assay

For analytical sensitivity and specificity of RLDT, ETEC strain H10407 (LT^+^ STh^+^ STp^+^) and *Shigella flexneri* 2a 2457T were used as ETEC and Shigella positive strains, respectively. These strains were obtained from the Walter Reed Army Institute of Research (WRAIR) and were previously used in our experimental challenge studies at JHU [18–20]. The naïve stool samples used were from donors negative for ETEC and Shigella. The strains were cultured in LB broth and incubated at 37°C for approximately 6–7 hours. The Colony Forming Units (CFU) was determined by optical density and quantitative plate counts from the culture. The naïve stool sample was aliquoted and spiked with 10-fold serially diluted cultures of ETEC or Shigella. The spiked stool samples and the naïve stool samples were processed for RLDT as described in Fig 1. To determine specificity, RLDT was tested using a range of positive and negative strains of *E*. *coli* and *Shigella spp*, as well as other enteric pathogens like *Vibrio cholerae*, *Campylobacter spp*, and *Salmonella typhi*.

The RLDT assay was evaluated for repeatability, reproducibility, accuracy, matrix inhibition, and linearity directly from spiked serially diluted stool samples using RLDT kit. The lyophilized RLDT LRT strips were also tested for stability at room temperature, 37°C and 42°C.

The sensitivity of ETEC and Shigella RLDT assay was also tested with purified DNA extracted from stool samples which were spiked with ETEC and Shigella (10^4^ to 10^0^ CFU/gm of stool) culture of known CFU. DNA was isolated from the spiked stool using a bead beater with 3-mm-diameter solid-glass beads (Sigma-Aldrich) and subsequently 0.1 mm zirconium beads (BIO-SPEC Inc.) to disrupt cells. The cell slurry was centrifuged at 16000 *g* for 1 min and the supernatant processed using the Qiagen QIAamp DNA stool extraction kit as described before [7]. DNA concentrations were measured using Nanodrop 1000 and 100ng of DNA was added to each tube of the RLDT LRT strips for 25μl RLDT reaction.

The naïve stool samples negative for ETEC and Shigella used in this study for spiking with respective pathogens, were obtained from the clinical study protocols [18,19] conducted under BB- IND 12,243 at the Johns Hopkins Bloomberg School of Public Health.

### Statistical analysis

The coefficient of variations (CV) was calculated as the ratio of the standard deviation to the mean (average). The linearity was determined by plotting the log TTR values against CFU/gm of stool, and the Pearson correlation coefficient was calculated. All graphs and statistical analysis were done with GraphPad, CA (version 9), and Stata Corp LLC (version 16) software.

## Results

### ETEC and Shigella detection using RLDT

Optimum TTR, sensitivity, and specific amplification were obtained when the reaction was performed at 71°C for 40 minutes. Analyzing the results considering TTR, RFU, specificity, and sensitivity, the threshold was set at 4000 RFU. All subsequent reactions were performed with these settings, unless noted.

**Fig 2:**
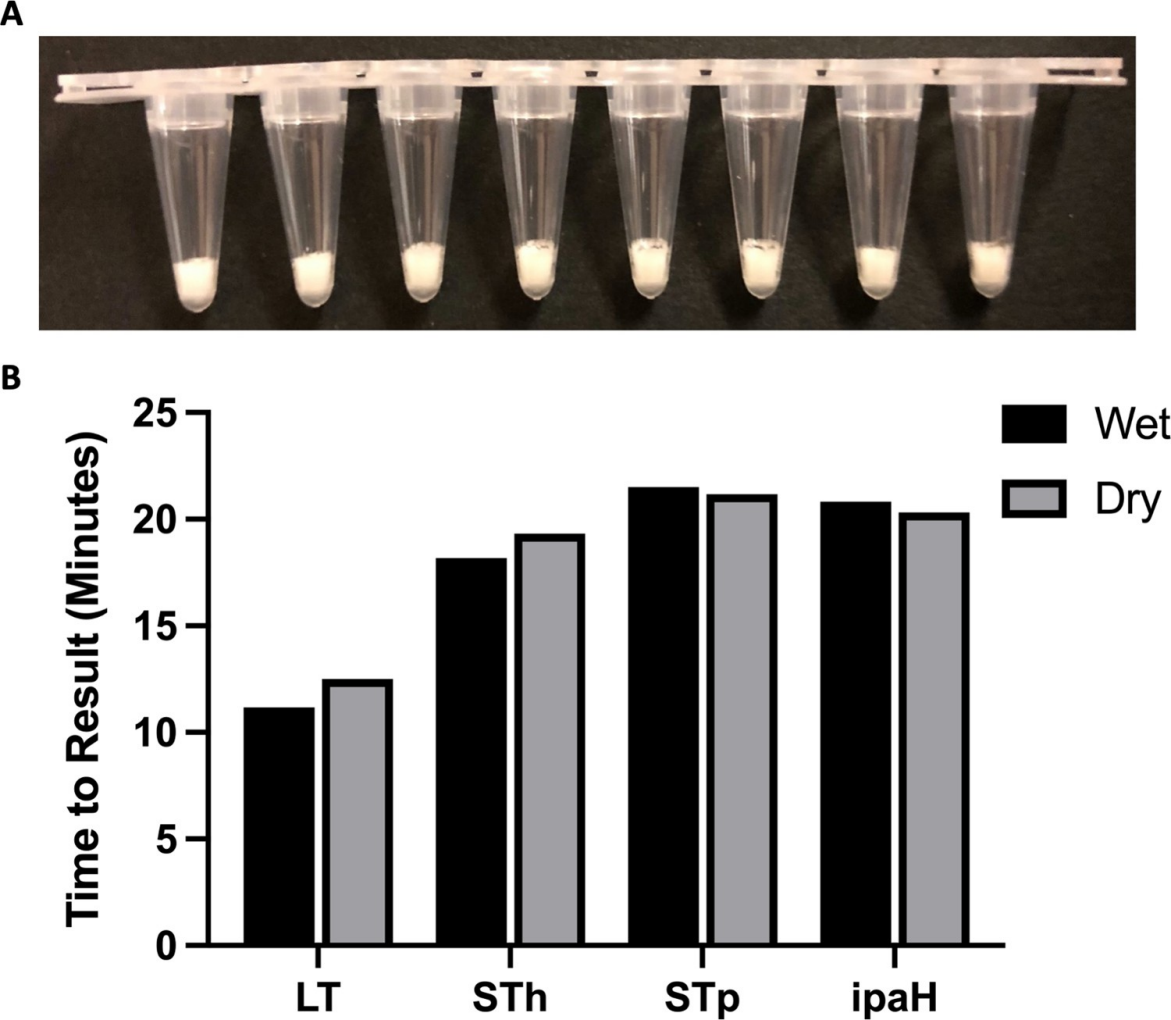
Dry formulation of RLDT LRTs. **2A** Appearance of RLDT lyophilized reaction tubes (LRT) strips. **2B** Time to result (TTR) for wet and dry formulations of ETEC and Shigella RLDT. LRT strips were tested using RLDT kit before (wet) and after lyophilization (dry). Black: Wet; Gray: Dry.

A dry formulation of the ETEC and Shigella RLDT reagents (Fig 2A) were compared with wet RLDT reagents for the detection of ETEC and Shigella target genes. No significant diminution of TTR or sensitivity was seen with the dried formulation compared to the wet preparation (Fig 2B).

To evaluate the stability of the dry RLDT formulations, the dry LRT strips were incubated at room temperature (~23°C), 37°C for 90 days and 42°C for 60 days and tested using the RLDT kit. Dry RLDT formulation were stable and functional at all the temperatures (S1 Fig). The TTRs were longer after two months but within 40 minutes. The RLDT stabilization process is further improved with optimized excipients and this improvement is currently undergoing further stability testing.

### Analytical performance of ETEC and Shigella RLDT kit

Results from all the RLDT kit analytical performance specification tests are described in S1 Table. The assay inhibitor control was used in every experiment. Positive results were defined when the amplification RFU reached the threshold within 40 minutes of the reaction, and the assay inhibitor control was positive.

**Fig 3:**
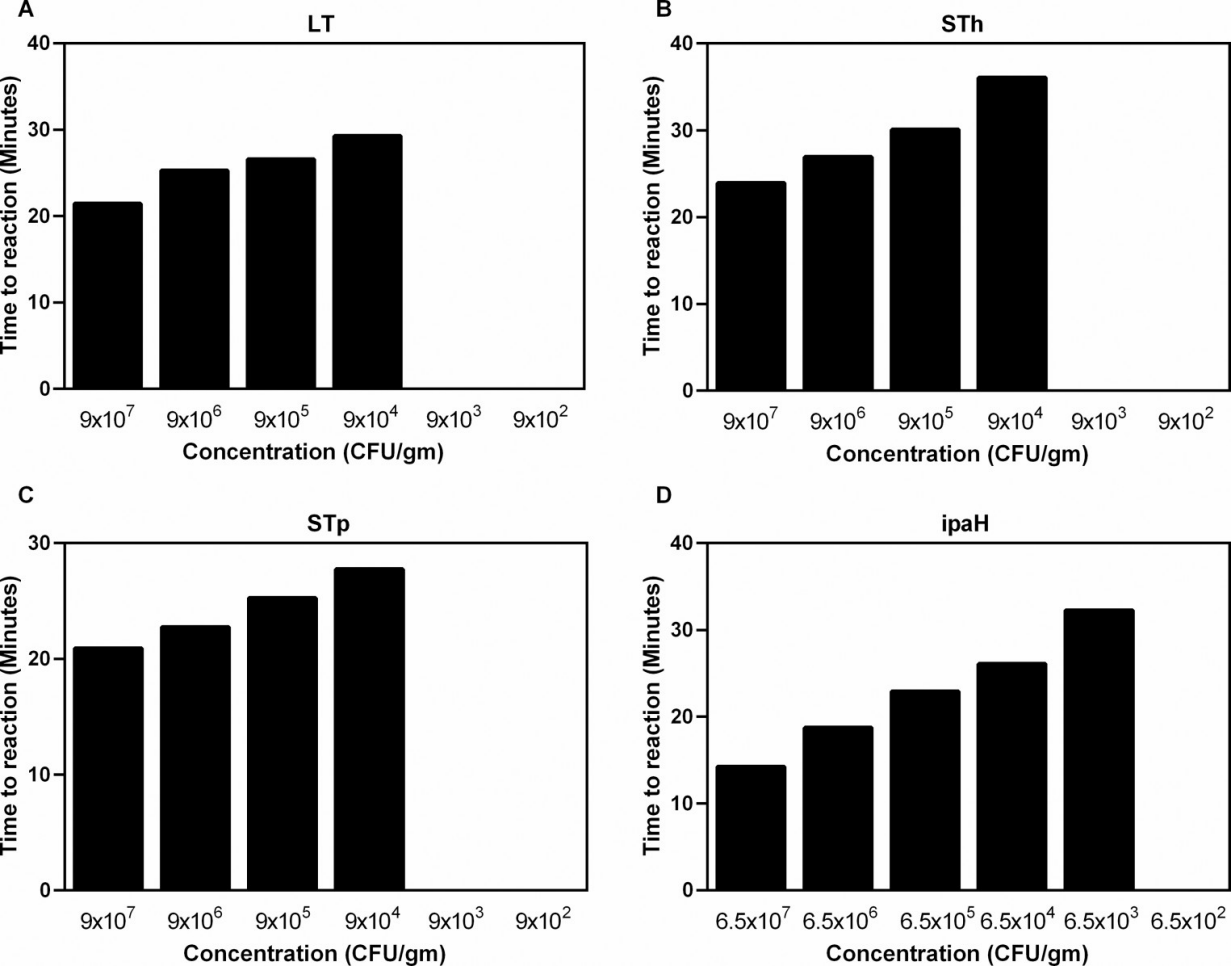
Lowest detection limit (LOD) of ETEC and Shigella RLDT directly from stool. LOD was determined against ETEC H10407 and *S*. *flexneri* 2a 2457T strains. Ten-fold serial dilutions of each strain was made in stool. The spiked stool samples were processed and tested with RLDT kit. A: ETEC LT gene; B: ETEC STh gene; C: ETEC STp gene and D: Shigella *ipaH* gene.

To determine the analytical sensitivity of RLDT, 10-fold serial dilutions (ranged from 10^2^ to 10^8^ CFU/gm of stool) of ETEC and Shigella spiked stool were run ten times using the RLDT kit. The lowest detection limit (LOD) was 9x10^4^ CFU/gm of stool for LT, STh, and STp and 6.5x10^3^ CFU/gm of stool for the *ipaH* gene (Fig 3), which corresponds to about 23 CFU and 1 CFU per 25uL reaction respectively for each pathogen within 40 minutes. LOD was defined as the lowest concentration at which the target could be detected in all the ten runs with these spiked samples.

Analytical specificity was evaluated with reference strains (Table 2). No amplification was observed with naive stool or with pathogens other than the targets, suggesting that the RLDT assay is specific to ETEC and Shigella. Analytical accuracy was evaluated with stool samples spiked with reference or well characterized strains. The accuracy (sensitivity and specificity) for detecting both ETEC and Shigella targets was 100%. The *ipaH* gene could detect *S*. *flexneri* 2a, *S*. *dysenteriae*, *S*. *sonnei*, and *S*. *boydii*. Specific target genes could be detected with RLDT when single stool sample spiked with multiple strains.

**Table 2:** List of strains used for the evaluation of the specificity of ETEC and Shigella RLDT.

Pathogen/Strain	Source	LT	STh	STp	*ipaH*
ETEC H10407	WRAIR	**+**	**+**	**+**	**-**
ETEC B7A	WRAIR	**+**	**+**	**+**	**-**
ETEC E24377A	WRAIR	**+**	**+**	**-**	**-**
*E*. *coli* 25922	ATCC	**-**	**-**	**-**	**-**
EPEC 335265–3	Bangladesh	**-**	**-**	**-**	**-**
CR112/C3/RS(C1)	Bangladesh	**-**	**-**	**-**	**-**
SP207X-141-2	Peru	**+**	**-**	**-**	**-**
SP076X-235-1	Peru	**+**	**-**	**+**	**-**
CR113/C9/RS(C1)	Bangladesh	**+**	**-**	**-**	**-**
CR100/C5/RS(C1)	Bangladesh	**+**	**+**	**-**	**-**
ETEC 335093–3	Bangladesh	**+**	**-**	**+**	**-**
ETEC 335140–1	Bangladesh	**-**	**+**	**+**	**-**
ETEC 335152–1	Bangladesh	**+**	**-**	**-**	**-**
*Shigella flexneri* 2a 2457T	WRAIR	**-**	**-**	**-**	**+**
*Shigella dysenteriae* AMC 43-A-1	ATCC	**-**	**-**	**-**	**+**
*Shigella boydii* AMC 4006	ATCC	**-**	**-**	**-**	**+**
*Shigella sonnei* 53G	WRAIR	**-**	**-**	**-**	**+**
*Vibrio cholera* O1 N16961	ATCC	**-**	**-**	**-**	**-**
*Vibrio cholera* O1 14035	ATCC	**-**	**-**	**-**	**-**
*Vibrio cholera* O139 51394	ATCC	**-**	**-**	**-**	**-**
*Campylobacter jejuni* 33291	ATCC	**-**	**-**	**-**	**-**
*Campylobacter coli* 33559	ATCC	**-**	**-**	**-**	**-**
*Salmonella enterica* serovar Typhimurium 700720DQ	ATCC	**-**	**-**	**-**	**-**
ETEC H10407 + *Shigella flexneri* 2a 2457T		**+**	**+**	**+**	**+**

Repeatability was tested with ten repeats of two samples, respectively spiked with a high (10^7^ CFU/gm of stool) and a low (10^5^ CFU/gm of stool) concentration of each target. Reproducibility was tested with ten identically spiked samples for each concentration, high and low, that were assayed over five days. Both dilutions met the criteria for positive results for all (100%) of the repeatability and reproducibility assays. The stool is a complex substrate, and the matrix can vary between samples. We tested matrix inhibition using three lots of stool from healthy donors spiked with ETEC and Shigella in stool. No difference was observed among the three lots for any of the target genes, and the results were as expected.

To understand if RLDT could be used as a semi-quantitative assay, we analyzed the linearity of the TTR values of ETEC and Shigella target genes using serially diluted spiked stool samples. Linearity was established by averaging the TTR over three runs and plotting the average results. The TTR increased consistently as the concentration of the bacteria in the samples decreased. The R^2^ linearity values were between 0.88 to 0.99; p<0.05 (S2 Fig). We also analyzed the %CV of the TTR values of ETEC and Shigella during repeatability and reproducibility experiments. The TTR values of the target genes had repeatability, within-run variance from 2.78% to 9.43% and reproducibility, between-run variance from 4.41% to 12.90% (S2 Table).

### Sensitivity of ETEC and Shigella RLDT kit from purified DNA

The sensitivity of ETEC and Shigella RLDT was also determined using purified DNA extracted from stool using bead beater and DNA purification kit. RLDT could detect ETEC and shigella genes from the purified DNA extracted from the lowest concentration of 10^2^ CFU/gm of stool within 40 minutes, which corresponds to ~1bacteria per 25μl of RLDT reaction.

### Optimization of RLDT kit from dried stool spots on filter paper

**Fig 4:**
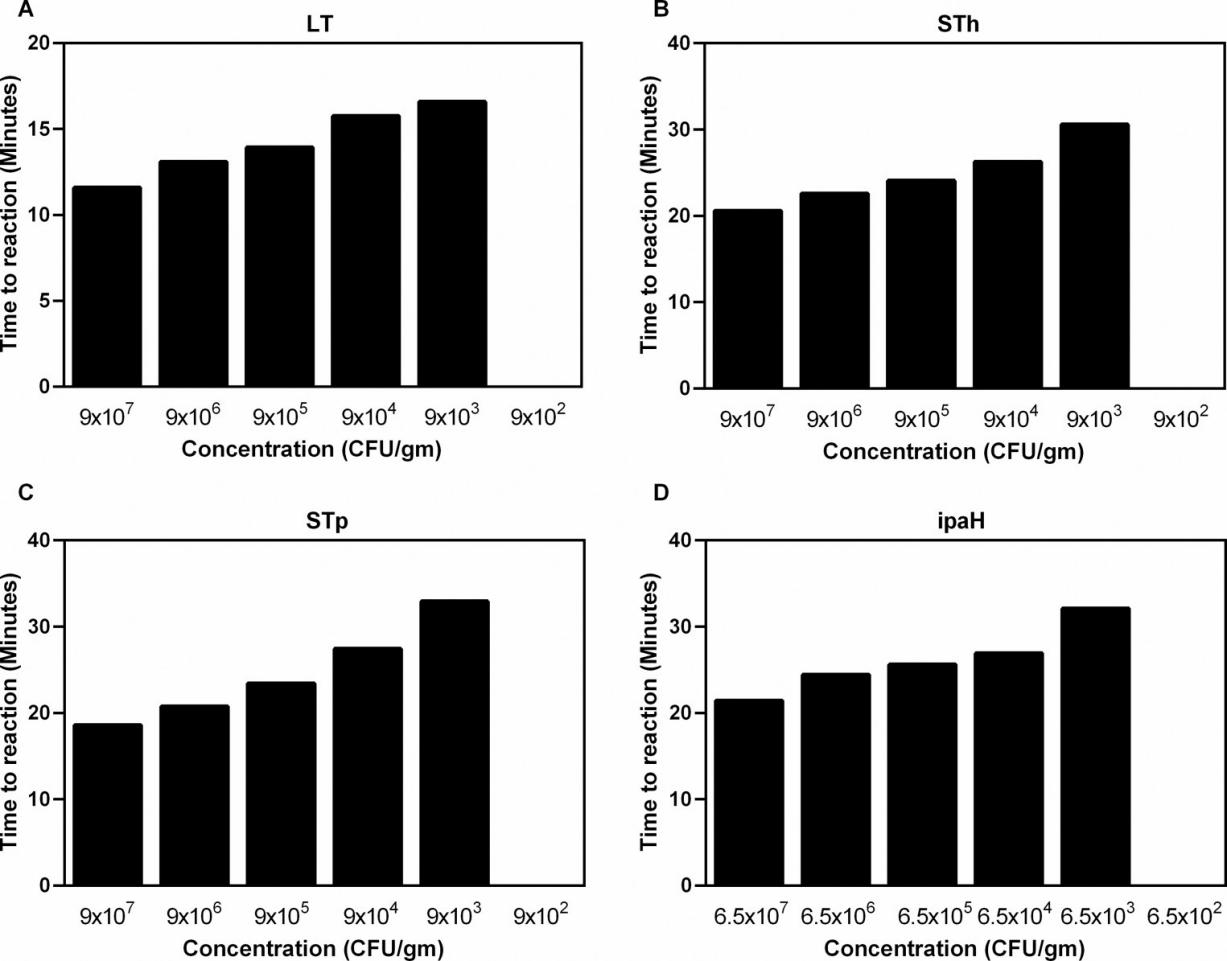
Lowest detection limit (LOD) of the ETEC and Shigella RLDT from dried stool on filter paper. LOD was determined against ETEC H10407 and *S*. *flexneri* 2a 2457T strains. Ten-fold serial dilutions of each strain was made in stool and 50μl each were spotted on filter paper. The stool spots were processed and tested with RLDT kit. A: ETEC LT gene; B: ETEC STh gene; C: ETEC STp gene and D: Shigella *ipaH* gene.

We tested if RLDT could be performed from dried stool on filter paper and determined LOD. Naïve stool samples spiked with serial dilutions of ETEC and Shigella culture were used to obtain concentrations of the pathogens 10^8^ to 10^2^ CFU/gm of stool. Fifty microliters of each of the spiked stool samples for ETEC and Shigella were placed on Whatman 903 Protein Saver Cards (Millipore, Sigma, MO, USA) and left to dry overnight. The following day, dried samples on the cards were cut out with sterile scissors, added to the SPT tube and was processed as described before directly from the stool (Fig 1). The LOD was 9x10^3^ CFU/gm of stool for ETEC genes, and 6.5x10^3^ for *ipaH* (Fig 4).

### Advantages of RLDT over current diagnostic assays of ETEC and Shigella

RLDT is performed directly from the stool samples with minimum treatment. Using the rapid sample preparation and dry formulation, the assay is simple. There is a minimum hands-on time of ~ 5 minutes for processing the sample and adding the lysates to the LRTs. The assay results are read as +/- using a battery-operated handheld reader. The RLDT kit can be stored at ambient temperature and thus can avoid maintaining a cold chain. The RLDT kit provides all the reagents and supplies required and could mitigate the constraints in obtaining reagents, primers, and plastics in LMICs. The LRTs are already filled with dry reagents and primers, avoids individual additions of each reagent, minimizing contaminations and simplify the process for the users. The assay is rapid, taking <50 minutes from stool to result. As six primers are used for detecting each target, RLDT has high specificity. RLDT only requires a heat block and a reader, which is optional. RLDT is comparatively inexpensive and costs ~ <$3 per target. The assay generates minimum biohazard wastes.

## Discussion

The novel RLDT for detecting ETEC and Shigella is a rapid and simple nucleic acid amplification-based diagnostic assay, which is suitable for laboratories and clinics at LMICs. Considering the goal for this assay is to provide point of care diagnostic for these bacterial enteropathogens in areas with limited access to adequate laboratory infrastructure, the RLDT assay needed to be optimized for maximum ease of use and ability for ambient storage. The stool is a challenging substrate for extraction of DNA and amplification because of various inhibitors, which can vary between samples. Therefore, in designing this test, we were confronted by the competing challenges making the sample processing procedure simple but at the same time sensitive and specific. We developed a simple and rapid sample preparation method directly from the stool, which resulted in a LOD of 10^4^ CFU/gm of stool for Shigella and 10^5^ CFU/gm of stool for ETEC respectively. These LODs are similar to those reported for detection of ETEC and Shigella genes using the TaqMan Array Card for enteropathogen detections [21] that has been used in the reanalysis of the samples from the Global Enteric Multicenter Study (GEMS) [13] and the multisite birth cohort study (MAL-ED) [22]. Of note, the TaqMan Array card uses purified DNA, and RLDT is performed directly from the stool.

To make the RLDT a dry format all reagents were lyophilized to improve their stability at room temperature. These modifications avoid handling of individual reagents as well as the requirement of maintaining a cold chain which makes RLDT more applicable to endemic countries where improved laboratories/clinics are often not available.

Like LAMP, the RLDT results can be read by the naked eye or using a UV illuminator. However, this may create end-user bias when using the assay in the field by technicians or the staff at the hospitals with minimum training. To address this difficulty, we preferred to use the handheld battery-powered fluorometer reader, which can read the results as positive or negative. This equipment adds additional cost to the assay, but it is only a one-time primary investment.

Since RLDT assay takes only ~50 minutes from stool to result and thus, ETEC and Shigella stool samples that are positive by RLDT can be cultured on the same day to isolate colonies for downstream characterization of the strains, using serotyping for both ETEC and Shigella, colonization factors typing of ETEC, antibiotic susceptibility testing and whole genome sequencing.

Although RLDT is a qualitative test, we observed a linear relation between the TTR of RLDT and CFU of the bacteria per gram of stool. Thus, the TTR in RLDT has the ability to semi-quantify the number of the target bacteria in the stool. In addition, depending on the user’s or study requirement, the reader could be programmed to set the cut-off TTR (corresponding to approximate CFU/copy numbers of the target bacteria in sample) to determine positive/negative results.

In recent years, several LAMP-based assays have been developed in the laboratories for diagnosis of infectious pathogens, including ETEC [17,23,24] and Shigella [25–29]. Stool is a complex sample to extract DNA and amplify because of the presence of inhibitors. The LAMP assays previously developed to detect enteric pathogens from stool are either from colonies isolated from culturing the stool or from purified DNA extracted from stool with commercial kits or a complex process that is not feasible at LMICs. In addition, these assays require maintaining a cold chain, which is difficult to achieve in these settings. RLDT has addressed these issues and adapted LAMP to be more applicable to the endemic settings where it is needed the most.

In conclusion, ETEC and Shigella RLDT described in this study has advantages, including rapid results, simple operating procedures, easy readout of the results, and a LOD equivalent to the detection of ETEC and Shigella using quantitative PCR. In addition, RLDT is electricity and cold chain-free. All these qualities make RLDT easy to scale up and appropriate to use in endemic settings.

We are currently leveraging the RLDT platform to develop diagnostic assays for other infectious diseases.

## Supporting information

S1 Table Performance specifications of ETEC and Shigella RLDT. (DOCX)

S2 Table RLDT Time to result (TTR) in repeatability and reproducibility analysis. (DOCX)

S1 Fig Stability of dry RLDT formulations. RLDT lyophilized reaction tube (LRT) strips were tested before (baseline) and after keeping at room temperature (~23°C), at 37°C and at 42°C for 2–3 months. The LRTs were tested with RLDT kit using spiked ETEC and Shigella cultures every month. A: ETEC LT gene; B: ETEC STh gene; C: ETEC STp gene and D: Shigella *ipaH* gene.
(TIF)

S2 Fig Linearity of RLDT Time to result (TTR) corresponds to the concentrations of ETEC or Shigella. Stool samples spiked with serially diluted ETEC and Shigella strains were tested with RLDT kit. Linearity was established by the average of log TTR values against CFU/gm of stool. Linear regression equation, Pearson correlation coefficient and p values are given in the table.
(TIF)
