# Supplementary material for: Development of a simple, rapid, and sensitive diagnostic assay for enterotoxigenic E. coli and Shigella spp applicable to endemic countries
Source: PLoS Negl Trop Dis. 2022 Jan 28;16(1):e0010180. doi: 10.1371/journal.pntd.0010180 (PMC8827434; doi:10.1371/journal.pntd.0010180)
Supplement: S1 Table — (DOCX) [file pntd.0010180.s001.docx]

**S1 Table. Performance specifications of ETEC and Shigella RLDT.**

| **Test performed** | **Test Method** | **Acceptance Criteria** | **Targets** | **Results (Pass/Fail)** |
| --- | --- | --- | --- | --- |
| Limit of Detection (LOD) | LOD is defined as number of copies per gram of stool that were consistently 100% detectable with 10 distinct extractions/amplifications. | Positive results are defined when the amplification reach the threshold within 40 minutes of the reaction and the assay inhibitor control is positive. | ETEC  LT  STh  STp  Shigella  *ipaH* | 9x10^4^CFU/gm of stool  6.5x10^3^ CFU/gm of stool |
| Repeatability | Tested with five repeats of two samples respectively spiked with a high (10^7^ CFU/gm of stool) and a low (10^5^ CFU/gm of stool) concentration of each ETEC and Shigella. |  | ETEC  LT  STh  STp  Shigella  *ipaH* | 20/20 (100%)  20/20 (100%)  20/20 (100%)  20/20 (100%) |
| Reproducibility | Tested with 10 identically spiked samples for each concentration (two concentrations, high and low, were interrogated) that were extracted and assayed over 5 days. |  | ETEC  LT  STh  STp  Shigella  *ipaH* | 20/20 (100%)  20/20 (100%)  20/20 (100%)  20/20 (100%) |
| Accuracy | RLDT was tested using reference samples as well as a range of positive and negative strains of enteric pathogens. |  | ETEC  LT  STh  STp  Shigella  *ipaH* | 100%  100%  100%  100% |
| Matrix Inhibition | Three different lots of stools from healthy donors were spiked with high and low concentrations of ETEC or Shigella and tested. |  | ETEC  LT  STh  STp  Shigella  *ipaH* | 6/6 (100%)  6/6 (100%)  6/6 (100%)  6/6 (100%) |
