# Supplementary material for: Development of a simple, rapid, and sensitive diagnostic assay for enterotoxigenic E. coli and Shigella spp applicable to endemic countries
Source: PLoS Negl Trop Dis. 2022 Jan 28;16(1):e0010180. doi: 10.1371/journal.pntd.0010180 (PMC8827434; doi:10.1371/journal.pntd.0010180)
Supplement: S2 Table — (DOCX) [file pntd.0010180.s002.docx]

**S2 Table.** **RLDT Time to result (TTR) in repeatability and reproducibility analysis.**

| **Targets** | **TTR CV (%)** | | | |
| --- | --- | --- | --- | --- |
|  | Repeatability | | Reproducibility | |
|  | 10^7^CFU/gm of stool | 10^5^CFU/gm of stool | 10^7^CFU/gm of stool | 10^5^CFU/gm of stool |
| LT | 4.06 | 2.78 | 6.94 | 12.38 |
| STh | 3.54 | 6.67 | 4.41 | 10.5 |
| STp | 3.34 | 8.51 | 7.71 | 12.54 |
| *ipaH* | 8.89 | 11.18 | 9.71 | 12.90 |
