# Supplementary figures and images for: Development of a simple, rapid, and sensitive diagnostic assay for enterotoxigenic E. coli and Shigella spp applicable to endemic countries
Source: PLoS Negl Trop Dis. 2022 Jan 28;16(1):e0010180. doi: 10.1371/journal.pntd.0010180 (PMC8827434; doi:10.1371/journal.pntd.0010180)

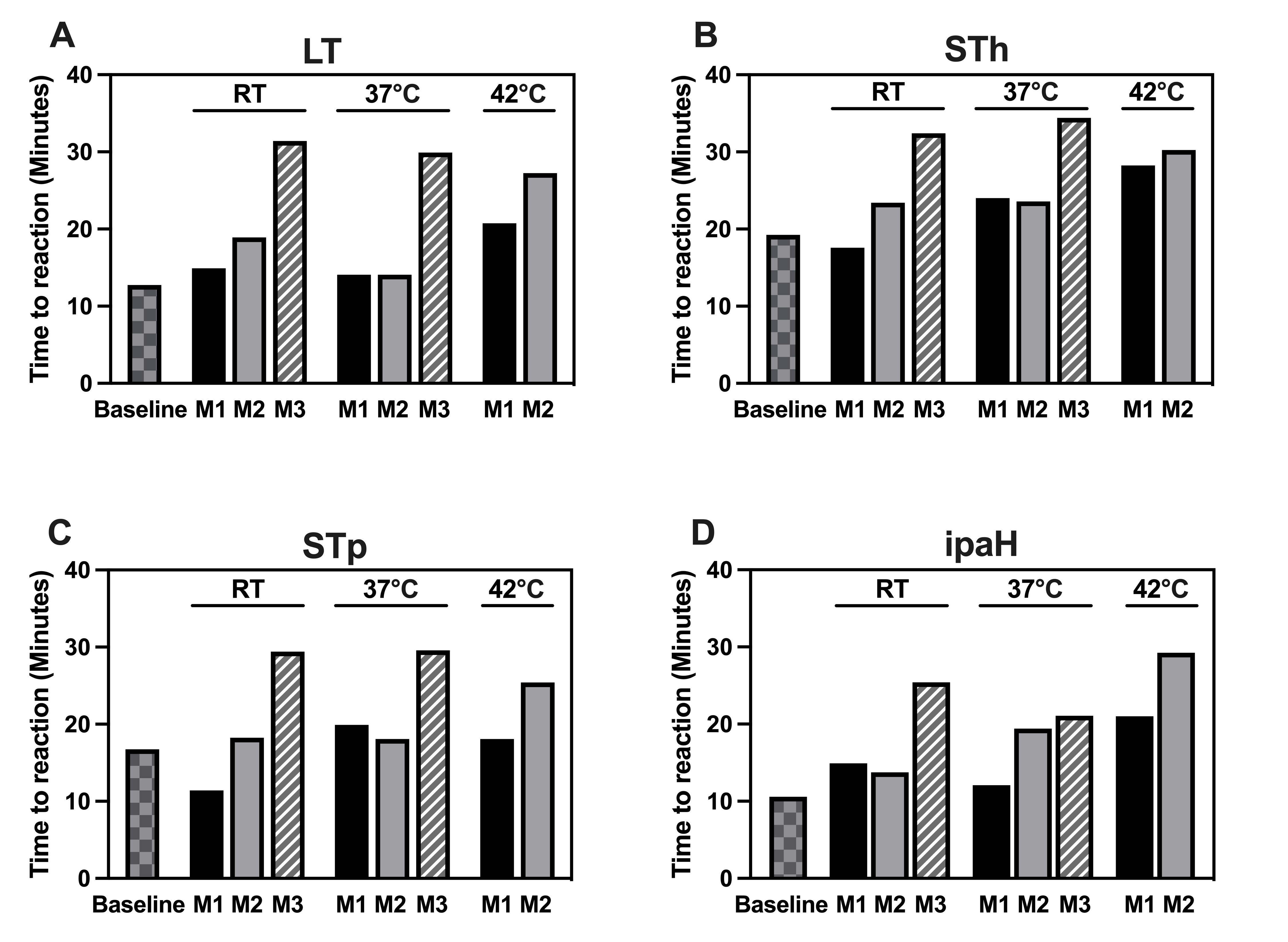

Supplement: S1 Fig — RLDT lyophilized reaction tube (LRT) strips were tested before (baseline) and after keeping at room temperature (~23°C), at 37°C and at 42°C for 2–3 months. The LRTs were tested with RLDT kit using spiked ETEC and Shigella cultures every month. A: ETEC LT gene; B: ETEC STh gene; C: ETEC STp gene and D: Shigella ipaH gene. (TIF) [file pntd.0010180.s003.tif]

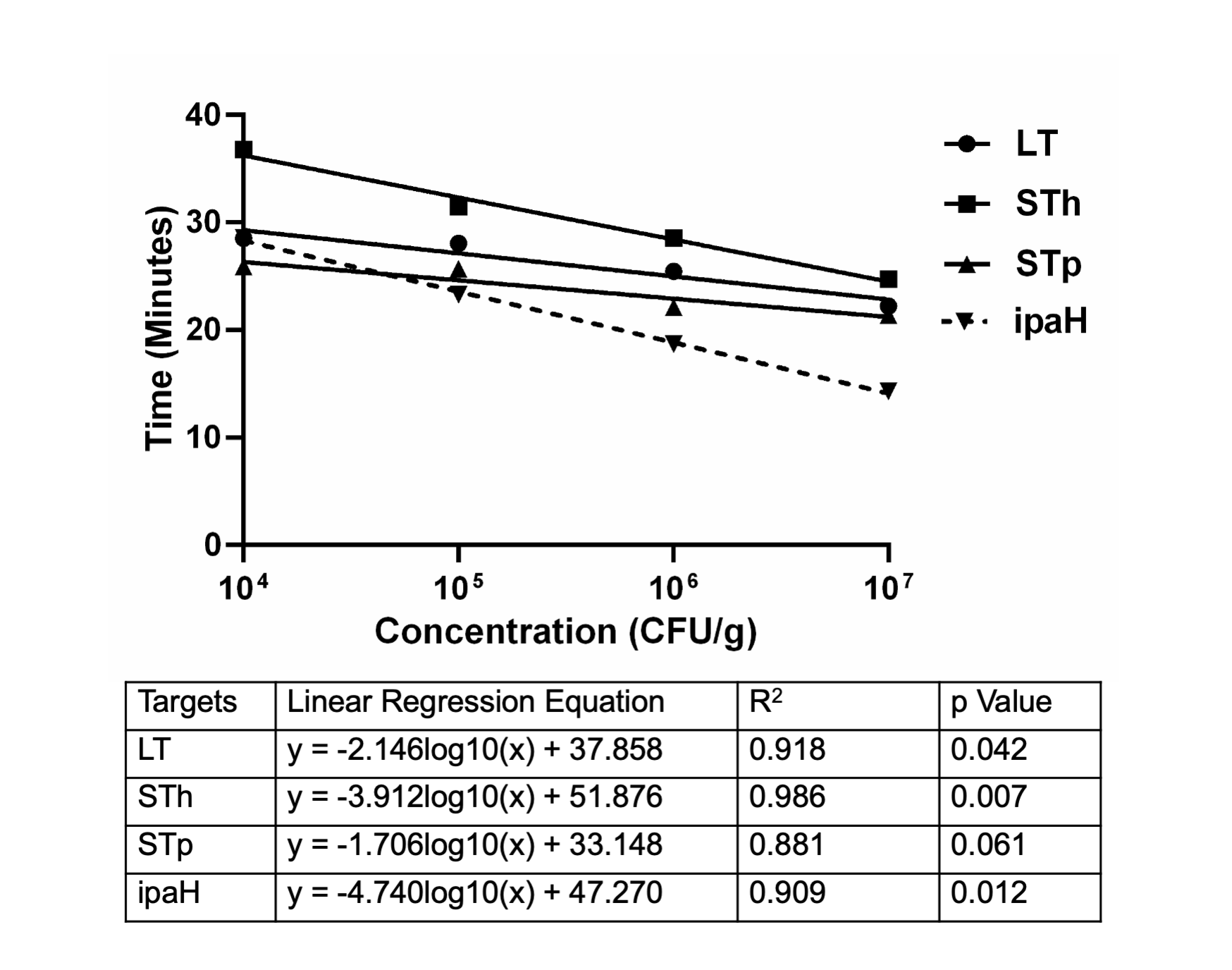

Supplement: S2 Fig — Stool samples spiked with serially diluted ETEC and Shigella strains were tested with RLDT kit. Linearity was established by the average of log TTR values against CFU/gm of stool. Linear regression equation, Pearson correlation coefficient and p values are given in the table. (TIF) [file pntd.0010180.s004.tif]
